# Genetic risk for alzheimer disease is distinct from genetic risk for amyloid deposition

**DOI:** 10.1002/ana.25530

**Published:** 2019-07-01

**Authors:** Ganna Leonenko, Maryam Shoai, Eftychia Bellou, Rebecca Sims, Julie Williams, John Hardy, Valentina Escott‐Price, the Alzheimer's Disease Neuroimaging Initiative

**Affiliations:** ^1^ Medical Research Council Centre for Neuropsychiatric Genetics and Genomics Cardiff University Cardiff; ^2^ Reta Lilla Research Laboratories, Department of Neurodegeneration and UK Dementia Research Institute University College London Institute of Neurology London; ^3^ UK Dementia Research Institute at Cardiff University Cardiff; ^4^ UK Dementia Research Institute at University College London London United Kingdom

## Abstract

**Objective:**

Alzheimer disease (AD) is the most common form of dementia and is responsible for a huge and growing health care burden in the developed and developing world. The polygenic risk score (PRS) approach has shown 75 to 84% prediction accuracy of identifying individuals with AD risk.

**Methods:**

In this study, we tested the prediction accuracy of AD, mild cognitive impairment (MCI), and amyloid deposition risks with PRS, including and excluding *APOE* genotypes in a large publicly available dataset with extensive phenotypic data, the Alzheimer's Disease Neuroimaging Initiative cohort. Among MCI individuals with amyloid‐positive status, we examined PRS prediction accuracy in those who converted to AD. In addition, we divided polygenic risk score by biological pathways and tested them independently for distinguishing between AD, MCI, and amyloid deposition.

**Results:**

We found that AD and MCI are predicted by both *APOE* genotype and PRS (area under the curve [AUC] = 0.82% and 68%, respectively). Amyloid deposition is predicted by *APOE* only (AUC = 79%). Further progression to AD of individuals with MCI and amyloid‐positive status is predicted by PRS over and above *APOE* (AUC = 67%). In pathway‐specific PRS analyses, the protein–lipid complex has the strongest association with AD and amyloid deposition even when genes in the *APOE* region were removed (*p* = 0.0055 and *p* = 0.0079, respectively).

**Interpretation:**

The results showed different pattern of *APOE* contribution in PRS risk predictions of AD/MCI and amyloid deposition. Our study suggests that *APOE* mostly contributes to amyloid accumulation and the PRS affects risk of further conversion to AD. ANN NEUROL 2019;86:427–435

Alzheimer disease (AD) is the most common form of dementia in elderly people and is a major health problem worldwide.^1^ The clinical diagnosis is typically characterized by progressive loss of memory and cognitive function. In the past decade, numerous relevant susceptibility loci, genes, and pathways have been identified^2^, ^3^, ^4^, ^5^, ^6^ that have improved the understanding of this complex disease. However, the risk for developing AD involves multiple genetic and environmental components, with the *APOE* genotype^7^ having the strongest genetic effect.^2^

Amyloid‐beta (Aβ) plays a key role in the pathogenesis of AD, but little is known about the process of its formation in the brain. Identification of the earliest pathological signature of AD requires longitudinal measurements of Aβ deposition in the brain by positron emission tomography (PET) or by measurements of Aβ reduction in cerebrospinal fluid (CSF). Although Aβ is necessary for the pathologic diagnosis of AD, it is not sufficient in itself to cause cognitive dysfunction and clinical AD. It has been shown that amyloid deposition has low specificity for predicting development of AD.^8^, ^9^

The preclinical stage of AD starts with mild impairment in cognitive domains (MCI) and includes a syndrome featuring relatively isolated memory deficits.^10^

In 2011, the National Institute on Aging and Alzheimer's Association created separate sets of diagnostic guidelines for the symptomatic or “clinical” stages of AD,^11^, ^12^ where AD represents the “disease” and “dementia” represents the clinical syndrome. Thus, a person may progress from MCI to dementia (due to AD), but both MCI and dementia cases may or may not be AD.

Studying individuals who develop MCI and then further progress to AD requires detailed longitudinal datasets. The Alzheimer's Disease Neuroimaging Initiative (ADNI) is a multicenter study designed to assess the utility of various biomarkers for detecting early changes associated with MCI and AD. It includes collection of neuroimaging data, clinical and cognitive assessments, and information on demographics and individual genetic profiles.

The polygenic risk score (PRS) approach aggregates the effects of multiple genetic markers identified through genome‐wide association studies (GWASs)^2^ and has shown great potential in identifying an individual's risk of developing AD.^13^, ^14^ A few studies have recently used AD PRS to predict mild cognitive functions and clinical MCI^15^; however, only one has suggested that PRS could identify MCI in middle aged adults^16^ more effectively than the *APOE* locus alone. The PRS approach has also been applied to biological pathways related to AD but was not more predictive than *APOE* alone.^17^ The implementation of polygenic hazard score (PHS; closely related to PRS^18^) analysis in the ADNI data showed that PHS is associated with AD biomarkers (CSF and PET) in individuals without AD,^19^ and that higher PHS was associated with greater rates of cognitive and clinical decline, even after controlling for *APOE* status^20^; however, its predictive value was not quantified.

In this study, we estimate the predictive accuracy of PRS differentiating (1) AD cases versus controls, (2) MCI cases versus controls, and (3) amyloid‐positive versus amyloid‐negative individuals. We also investigate whether (4) the AD PRS can predict individuals with MCI who will progress to AD and those who will remain with MCI, with positive amyloid deposition.

Recently, GWASs and exome/genome sequencing have implicated, with varying degrees of confidence, lipid metabolism, the innate immune system, and endosomal vesicle recycling in late onset AD pathogenesis.^21^, ^22^ Therefore, we also examined the pathway‐specific PRS association using these recently identified pathways^6^ related to AD risk.

## Materials and Methods

### *ADNI: Setting/Clinical Description*

Data used in the preparation of this article were obtained from ADNI, a publicly available database (https://adni.loni.usc.edu). The primary goal of ADNI has been to test whether serial magnetic resonance imaging (MRI), PET, other biological markers, and clinical and neuropsychological assessment can be combined to measure the progression of MCI and early AD. The data were collected for about 900 individuals between ages 55 and 90 years. The initial 5‐year study (ADNI1) followed participants for 2 to 3 years, with repeated imaging scans and psychometric measurements every 6 or 12 months. All ADNI participants provided written informed consent. The ADNI project was extended as the ADNI‐GO and ADNI2, studies with a proportion of new and original ADNI1 participants.

Clinical diagnosis and genetic information were available for 770 individuals from the ADNI1, ADNI‐GO, and ADNI2 studies. Longitudinal data contained information about clinical assessments from the first visit (baseline) to the latest available visit, with mean follow‐up time of approximately 5 years. Details of the ADNI design, participant recruitment, clinical testing, and additional methods have been previously reported elsewhere.^23^, ^24^

Table 1 shows the classification of diagnosis and number of individuals whose diagnosis remained stable during the study. It also presents the diagnostic categories and the numbers of individuals within those diagnostic categories at the latest assessment, which were used for the analyses.

**Table 1:** Clinical Classification of Diagnosis in ADNI Dataset

Diagnosis Description	Samples with Diagnosis at the First Time Point, n	Samples with Diagnosis at the Last Time Point, n	Samples Stable over Time, n	Usage for Analysis
Stable control to control	262	224	200	Controls
Stable MCI to MCI	459	289	267	MCI
Stable AD to AD	47	174	46	AD
Conversion control to MCI	0	20	0	Exclude
Conversion MCI to AD	1	50	0	MCI
Conversion MCI to control	1	8	0	Exclude
Conversion AD to MCI	0	5	0	MCI

Diagnosis description–classification of clinical diagnosis made for each participant and each time point. Second column shows number of participants with baseline diagnosis. Third column shows number of participants at the last point of diagnosis. Fourth column shows number of participants who did not change their diagnosis at the last assessment from baseline diagnosis. Last column shows clinical classification of individuals based on the last available diagnosis for our analyses.

AD = Alzheimer disease; ADNI = Alzheimer's Disease Neuroimaging Initiative; MCI = mild cognitive impairment.

To assess amyloid deposition, the latest MRI PET scans from 663 participants were used in the analysis (AV45 ligand threshold of 1.11). In this study, we used the individuals’ diagnosis at the latest point of assessment. We then tested whether AD PRSs were associated with AD, MCI, and amyloid status in 3 main analyses: (1) AD versus controls, (2) MCI versus controls, and (3) amyloid‐positive versus amyloid‐negative status (Table 2).

**Table 2:** ADNI Phenotypes and PET Amyloid Status

	Samples, n	n (% of MCI)	n (% of AD)	n (% of controls)
Amyloid positive	357	162 (47%)	120 (69%)	65 (29%)
Amyloid negative	304	148 (43%)	18 (10%)	128 (57%)
NA	89	34 (10%)	36 (21%)	31 (14%)
All samples	770	344	174	224

Shows number of individuals with positive/negative amyloid for clinically diagnosed samples (MCI, AD, and controls).

AD = Alzheimer disease; ADNI = Alzheimer's Disease Neuroimaging Initiative; MCI = mild cognitive impairment; NA = not available; PET = positron emission tomography.

### *ADNI: Genotyping and Quality Control*

A total of 770 samples from ADNI1/GO/2 set were whole‐genome sequenced (WGS) and genotyped using the Illumina (San Diego, CA) Omni 2.5M BeadChip (42,732,452 variants). WGS calls were made using the Broad Institute best practices (BWA & GATK HaplotypeCaller).

Basic quality control checks were performed using standard procedure.^25^ Single nucleotide polymorphisms (SNPs) were excluded where genotype missingness was >0.02, Hardy–Weinberg equilibrium *p* value was <1e‐6, and SNP minor allele frequency was <0.01. This retained 7,808,548 SNPs for the analyses. Matching those SNPs with the latest publicly available GWAS AD summary statistics^2^ reduced that number to 5,771,686.

### *Generating PRS*

Generation of PRS requires 2 independent datasets: summary statistics of association with AD in a discovery sample; and a test sample, which is independent of the discovery sample and contains genotypes for each individual.^26^ As the discovery sample, we used summary statistics from the powerful GWAS (17,008 AD cases and 37,154 controls) of the International Genomics of Alzheimer's Project (IGAP Stage 1).^2^ PRSs were generated using SNPs with AD association *p* ≤ 0.5 in the IGAP dataset, as it has been reported as having the best prediction accuracy.^13^ The SNPs were then linkage disequilibrium (LD) pruned (*r* ^2^ = 0.1 and 1,000kb window), keeping the SNPs most associated with AD. The number of SNPs after the LD pruning was 162,957. We included *APOE* ε2 and ε4 allele genotypes directly into the PRS with effect sizes B = −1.04 and B = 1.55 for ε2 and ε4, respectively, calculated in the ADNI data, while excluding the *APOE* region (chromosome 19:44,400–19:46,500kb).^13^ Prior to all analyses, the PRSs were adjusted for the 8 principal components and then standardized.

A total of 441 ADNI participants were part of original IGAP summary statistics.^2^ To overcome a potential bias in PRS analysis due to overlapping samples, we used a simulation approach we previously described.^14^ In brief, first we assessed the variation in the SNPs’ effect sizes using 1,000 simulations when randomly excluding 266 cases and 173 controls (matching the numbers of overlapping samples). The variation in the IGAP effect sizes due to the overlap was estimated in terms of standard deviation (SD_IGAP_ = 0.053) from the mean (ie, the original IGAP SNP beta‐coefficient [Beta_IGAP_]). Then, new IGAP genome‐wide summary statistics were simulated 10,000 times with adjusted effect sizes (Beta_adjusted_) and *p* values for each SNP. Beta_adjusted_ was sampled from a normal distribution with mean = Beta_IGAP_ and SD = 0.053*SE_IGAP_; *p* values_adjusted_ were redefined accordingly. At each simulation, SNPs were reselected and repruned based on LD *r* ^2^ = 0.1 and 1,000kb window. The prediction accuracies (areas under the receiver operator curve [AUCs]) reported in the Results section are presented as means from these 10,000 simulations.

### *Genome‐wide and Pathway‐Specific PRS Predictions*

Initially, we tested whether PRSs are associated with AD risk (AD cases vs controls) in the ADNI dataset. Then we assessed whether the AD PRS can distinguish individuals with MCI from cognitively normal controls and amyloid‐positive from amyloid‐negative individuals. Finally, we assessed whether PRS can predict AD risk over and above *APOE* in MCI individuals who have had positive amyloid deposition (to be precise, the MCI individuals who converted to AD between the baseline and final time of assessment vs nonconverters). All analyses were performed using logistic regression models with the following predictors: (1) *APOE* (ε2 + ε4), (2) PRS without *APOE*, and (3) full PRS model (predictors 1 and 2 together). Gender and age were used as covariates in all analyses. We tested whether the PRS significantly improves the model fit over and above *APOE* alone with the anova() function in R. We report the accuracy of the models in terms of AUC. In addition, we calculated PRS prediction accuracies (AUCs) in the extremes of PRS distribution for individuals whose PRS score was greater or smaller than ±1.5 SD from the PRS mean.

For the pathway‐specific analyses, we chose the latest published 9 pathways that have been reported as playing a role in AD pathogenesis, namely (1) protein–lipid complex assembly, (2) regulation of beta‐amyloid formation, (3) protein–lipid complex, (4) regulation of amyloid precursor protein catabolic process, (5) reverse cholesterol transport, (6) protein–lipid complex subunit organization, (7) plasma lipoprotein particle assembly, (8) tau protein binding, and (9) activation of immune response.^6^ Finally, to quantify the proportion of variance that remains unexplained by the pathways together, we calculated and tested PRS for the whole genome excluding these 9 pathways.

Pathway‐specific PRSs were generated in the ADNI dataset for each individual as described above with and without the *APOE* region. The PRSs in this case were adjusted not only for 8 principal components but also for age and gender and then standardized.

The results were considered significant if the resulting *p* value was ≤1.85 × 10^−3^ = 0.05/(3 scenarios × 9 pathways), corresponding to the Bonferroni correction for multiple comparisons.

## Results

**Figure 1:**
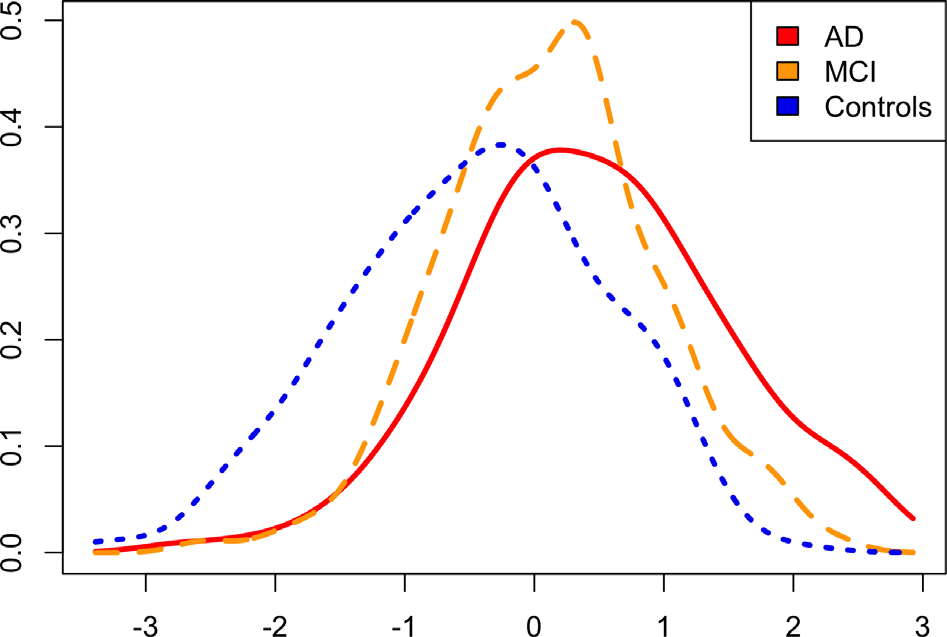
Density plots of polygenic risk score (PRS) for Alzheimer disease (AD), mild cognitive impairment (MCI), and cognitively normal participants. Standardized individual PRS scores for 3 phenotypes are shown (AD, MCI, and controls).

The prediction accuracy of AD cases (n = 174) versus controls (n = 224) at the last assessment point was AUC_*APOE*_ = 76% and AUC_PRS_ = 75%, for *APOE* alone and for PRS without *APOE*, respectively (Table 3, first row). The best prediction accuracy (AUC_FULL_ = 82%) was achieved with the full model, which includes both *APOE* and PRS. An analysis of variance test (last column of Table 3) confirmed that PRS significantly improves the prediction accuracy of the model over and above *APOE* (*p* = 1.7 × 10^−13^). A similar pattern of results was observed when we compared MCI individuals at the last point of assessment (n = 344; see Table 1 for details) with controls; however, the accuracy was reduced (AUC_*APOE*_ = 62%, AUC_FULL_ = 68%). Again, PRS significantly improves the prediction accuracy of MCI risk over and above *APOE* (*p* = 2.5 × 10^−11^). Figure 1 shows standardized density plots of polygenic risk scores in AD cases (red line), controls (blue line), and MCI (orange line), where the mean of the PRS for the latter is between the means of the PRS for AD cases and controls. Interestingly, the results for prediction of amyloid deposition by PRS follows a different pattern; *APOE* alone significantly predicted amyloid deposition with AUC_*APOE*_ = 76%, and PRS did not improve the prediction accuracy further.

**Table 3:** PRS and *APOE* Predictions of AD/MCI/Controls/Amyloid Phenotypes in ADNI

Model	Statistical Characteristics	AD vs Controls, n = 174/224	MCI vs Controls, n = 344/224	Amyloid Positive vs Amyloid Negative, n = 357/304
*APOE*	Beta^1^, ^3^, ^4^ [SE]	0.99 [0.13], −0.58 [0.22], 0.03 [0.01]	0.3 [0.1], −0.5 [0.17], −0.02 [0.01]	1.08 [0.01], 0.2 [0.17], 0.04 [0.01]
	*p*	1.06e‐18	9.6e‐5	<2.2e‐16
	AUC^a^/AUC^b^	0.72/0.76	0.58/0.62	0.72/0.76
PRS (*p* < 0.5) without *APOE*	Beta^2^, ^3^, ^4^ [SE]	0.93 [0.12], −0.7 [0.2], 0.016 [0.015]	0.68 [0.1], −0.47 [0.18], −0.007 [0.01]	0.3 [0.08], 0.13 [0.16], 0.023 [0.01]
	*p*	2.7e‐18	6.e‐12	1.4e‐3
	AUC^a^/AUC^b^	0.74/0.75	0.66/0.67	0.58/0.58
Full PRS model	Beta^1^, ^2^, ^3^, ^4^ [SE]	0.93 [0.13], 0.88 [0.13], −0.63 [0.24], 0.04 [0.02]	0.26 [0.1], 0.66 [0.1], −0.47 [0.18], −0.002 [0.01]	1.06 [0.1], 0.22 [0.09], 0.22 [0.17], 0.05 [0.01]
	*p*	1.9e‐30	1.1e‐12	2.3e‐29
	AUC^a^/AUC^b^	0.81/0.82	0.67/0.68	0.75/0.76
ANOVA *p* (PRS above *APOE*)		1.7e‐13	1.8e‐10	0.038

Beta^1^ = beta(e2 + e4), Beta^2^ = beta(PRS), Beta^3^ = beta(sex), Beta^4^ = beta(age). First column shows 3 scenarios where PRS predictions were made: *APOE* alone, PRS without *APOE*, and full model (*APOE* plus PRS [*p* < 0.5]). Second column shows statistical characteristics that were calculated for each model; these include effect size (beta) with SE, *p* values, and AUC (with and without gender and age) and *p* value of significance of PRS above *APOE* model. Columns 3–5 represent 3 analyses with number of samples where different models were tested.

a AUC without taking gender and age into account.

b AUC where gender and age were used as predictors.

AD = Alzheimer disease; ADNI = Alzheimer's Disease Neuroimaging Initiative; ANOVA = analysis of variance; AUC = area under the curve; MCI = mild cognitive impairment; PRS = polygenic risk score; SE = standard error.

When we tested the full PRS model for prediction of individuals at the extremes of polygenic score distribution (±1.5 SD from the PRS mean), the prediction accuracy as expected increased (AUC = 94% for AD vs controls and AUC = 91% for MCI vs controls).

**Figure 2:**
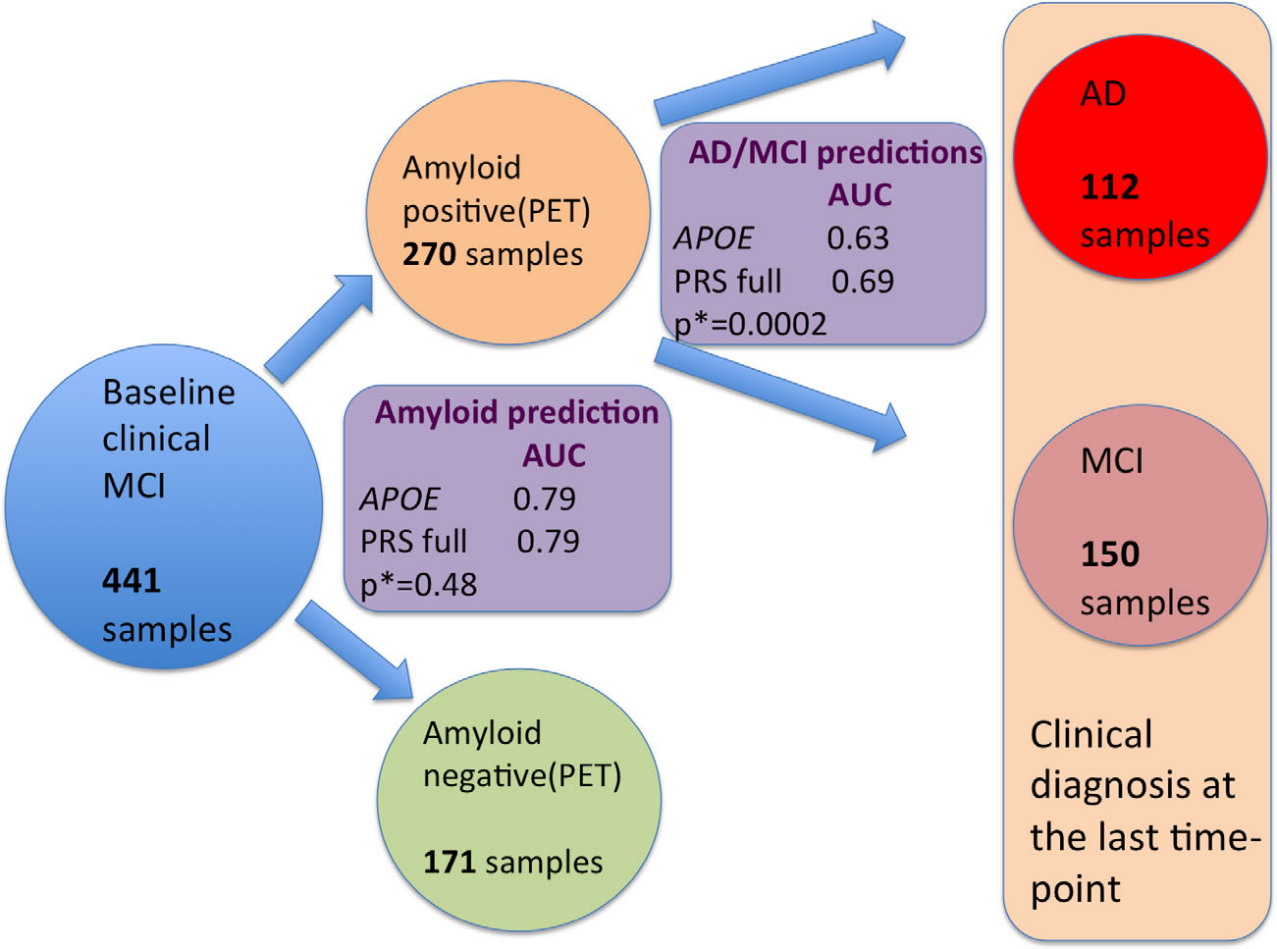
Diagram of prediction of amyloid deposition and further prediction of conversion of mild cognitive impairment (MCI) individuals to Alzheimer disease (AD) in the sample that was first clinically diagnosed with MCI using *APOE* and AD polygenic risk score (PRS). PRS predictions were first made for individuals who had baseline diagnosis of MCI. *APOE* alone and the full PRS model were used to predict amyloid deposition. The same models were used to predict which MCI individuals would convert to AD versus those individuals who had an MCI diagnosis using the latest clinical diagnosis. AUC = area under the curve; PET = positron emission tomography.

We tested whether the PRS can predict progression to AD in individuals with MCI. Of 459 individuals with MCI at the baseline assessment, 441 had known amyloid deposition status (270 were amyloid‐positive and 171 were amyloid‐negative). The prediction accuracy of amyloid deposition in this subsample was AUC_*APOE*_ = 79% by *APOE* alone and PRS did not improve the prediction accuracy (*p* = 0.48; Fig 2). Of 270 amyloid‐positive individuals, 112 have progressed to AD and 150 individuals remained MCI as of the last point of assessment. In this case, PRS did predict AD progression (AUC_*APOE*_ = 63% and AUC_FULL_ = 69%), significantly improving the prediction over and above *APOE* (*p* = 0.0002; see Fig 2).

**Figure 3:**
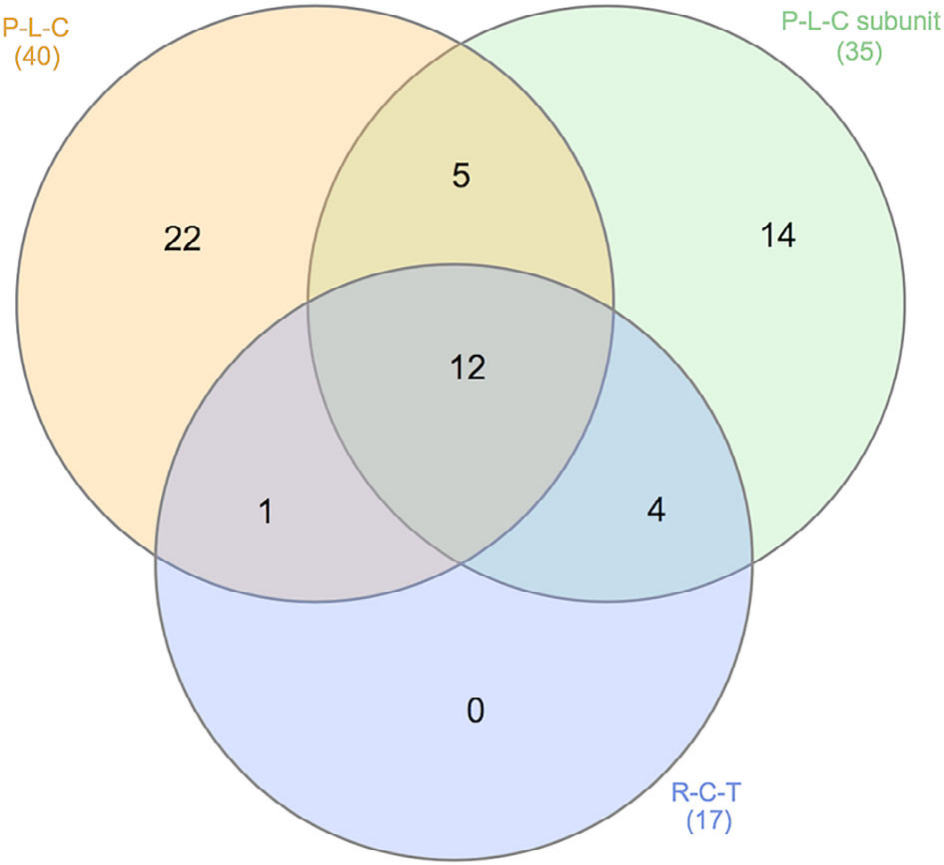
Overlap between 3 pathways: (1) protein–lipid complex (P‐L‐C; 40 genes), (2) protein–lipid complex subunit organization (35 genes), and (3) reverse cholesterol transport (R‐C‐T; 17 genes).

Finally, we calculated pathway‐specific PRSs and tested them for association with risk for AD, MCI, and amyloid deposition. The results are presented in Table 4. The majority of pathways were significantly associated with AD risk; however, this association was mostly driven by the *APOE* region. Two pathways (protein–lipid complex, protein–lipid complex subunit) remained significant after removing genes in the *APOE* region. When we excluded all pathways from the whole genome PRS, we observed that a substantial part of variance still remained unexplained (*p* = 2.2 × 10^−14^; last row of Table 4). Comparing amyloid‐positive versus amyloid‐negative individuals, the same 2 protein‐lipid–related pathways and additionally reverse cholesterol transport were significant after removing genes in the *APOE* region. The association results of the 9 pathways’ PRS with MCI risk were nominally significant for all pathways, and the association was mostly attributed to *APOE*. This clearly demonstrates that the pathways that contain the *APOE* region are strong predictors of amyloid deposition. Protein–lipid complex has shown the strongest association with AD and amyloid deposition risk in all the analyses. The overlap of genes in the 3 pathways above is presented in Figure 3.^27^

**Table 4:** Prediction of AD and Amyloid Deposition Risk with Pathway‐Specific PRSs

Pathways	Genes, n	AD (n = 174) vs Controls (n = 224)			Amyloid Positive (n = 357) vs Negative (n = 304)		
		Beta	*p*	*p* (no *APOE* region)	Beta	*p*	*p* (no *APOE* region)
Protein–lipid complex assembly	20	0.87	3e‐13	0.35	0.94	4e‐21	0.4
Regulation of beta‐amyloid formation	10	0.79	1.1e‐11	0.09	0.81	8.9e‐17	0.47
Protein‐lipid complex	40	0.91	8.14e‐14	5.5e‐3	0.96	1.8e‐21	7.9e‐3
Regulation of amyloid precursor protein catabolic process	12	0.79	1.1e‐11	0.09	0.81	9.6e‐17	0.49
Tau protein binding	11	0.77	3.1e‐11	0.39	0.82	4.8e‐17	0.6
Reverse cholesterol transport	17	0.84	2.4e‐12	0.07	0.93	2.1e‐19	0.03
Protein–lipid complex subunit organization	35	0.92	8.e‐14	0.03	0.97	9.67e‐22	0.03
Plasma lipoprotein particle assembly	18	0.89	2e‐13	0.66	0.94	3.6e‐21	0.98
Activation of immune response	432	0.18	0.06	0.06	0.21	6.8e‐3	0.01
Whole genome without all pathways	–	0.93	2.2e‐14	–	0.38	9.1e‐6	–

First column shows names of pathways that were analyzed. Second column shows number of genes in each pathway. PRS pathway‐specific effect sizes with *p* values and *p* values (no *APOE* region) of the models are presented in columns 3–8 for AD vs controls and amyloid deposition status.

AD = Alzheimer disease; PRS = polygenic risk score.

Finally, we tested these pathways’ PRS for association with amyloid deposition in individuals with MCI and with their further progression to AD when their amyloid deposition status was positive. We found that protein–lipid complex, protein–lipid complex subunit organization, and reverse cholesterol transport pathways are also associated with amyloid deposition even after exclusion of the *APOE* region (Table 5, 4th column).

**Table 5:** Prediction of Amyloid Deposition in Individuals with MCI and of Progression to AD in Individuals with MCI and Positive Amyloid Deposition with Pathway‐Specific PRSs

Pathways	Amyloid Positive (n = 270) vs Amyloid Negative (n = 171)			MCI and Amyloid Positive (AD [n = 112] vs MCI [n = 150])		
	Beta	*p*	*p* (no *APOE* region)	Beta	*p*	*p* (no *APOE* region)
Protein–lipid complex assembly	1.11	1.92e‐17	0.2	0.48	2.7e‐4	0.81
Regulation of beta‐amyloid formation	0.95	7.6e‐14	0.2	0.30	9e‐3	0.11
Protein–lipid complex	1.12	1.1e‐17	3.1e‐3	0.51	1.5e‐4	0.23
Regulation of amyloid precursor protein catabolic process	0.95	8.4e‐14	0.2	0.3	9.4e‐3	0.12
Tau protein binding	0.99	2.2e‐14	0.2	0.2	0.08	0.24
Reverse cholesterol transport	1.05	1.9e‐15	0.03	0.31	0.01	0.24
Protein–lipid complex subunit organization	1.1	1.2e‐17	0.05	0.51	1.9e‐4	0.64
Plasma lipoprotein particle assembly	1.09	3.4e‐17	0.9	0.51	1e‐4	0.31
Activation of immune response	0.18	0.068	0.09	0.08	0.54	0.76
Whole genome PRS without pathways	0.36	2.1e‐3	–	0.6	8.8e‐5	–

First column shows names of pathways that were analyzed. PRS pathway‐specific effect sizes with *p* values and *p* values (no *APOE* region) of the models are presented in columns 2–7.

AD = Alzheimer disease; MCI = mild cognitive impairment; PRS = polygenic risk score.

## Discussion

The pathological process related to AD starts long before clinical onset and lasts approximately 15 to 20 years^28^ It is widely believed that identifying individuals who have high risk of AD earlier is essential for therapeutic strategies for AD prevention and intervention.^29^ Due to the diagnostic heterogeneity of MCI and different length of follow‐up assessments, the conversion rate to AD or other types of dementia varies widely over different studies.^30^, ^31^ Identifying individuals with MCI and monitoring them through biomarker measurements should provide a better understanding of the process of progression from MCI to AD. Although there is no generally accepted diagnostic criteria that specifies MCI individuals who will convert to AD, it is notable that an increase in amyloid plaques that starts many years before clinical symptoms appear plays an important role in brain degenerative changes.

A reasonable prediction accuracy can be achieved with a PRS approach that uses genetic profile information and relates it to AD risk.^13^, ^14^ The PRS and its modifications have been assessed for association with AD and AD‐related phenotypes in a number of studies; however, the reported prediction accuracies have not been entirely consistent. In this study, we examined prediction accuracy that can be achieved with *APOE* alone and with the full PRS model differentiating between AD, MCI, controls, and amyloid status.

We have shown that the best prediction accuracy can be achieved with the PRS that includes *APOE* for both AD versus controls and MCI versus controls analyses (AUC = 82% and AUC = 68%, respectively). In both analyses, the PRS improves the prediction accuracy by about 8 to 9% compared to *APOE* alone, which replicates the analyses in independent datasets published elsewhere.^13^, ^14^, ^16^ Of course, GWASs indicate that *APOE* is the strongest risk factor and other common genetic variants have smaller effect sizes. However, the *APOE* region explains ~5% of SNP heritability, whereas the whole genome explains ~24%.^32^ In addition, PRS prediction accuracy shows a substantial increase in AUC, which makes the PRS potentially clinically useful for disease risk prediction. Furthermore, AD GWAS risk loci have greatly expanded our understanding of the disease mechanisms.

As expected, the accuracy of MCI prediction is lower than AD, which can be explained by the inclusion of a subset of MCI individuals who will not develop AD. For individuals with extreme PRS, the AUC reaches 90% and above for both AD and MCI.

The prediction of amyloid deposition showed a different pattern. In the whole sample, the prediction accuracy with *APOE* alone was 76% and the PRS did not improve the accuracy any further (AUC remained 76%). Similar results were obtained when we tested the prediction accuracy of amyloid deposition in individuals with MCI. However, when we looked at individuals who have already had positive amyloid deposition and attempted to predict their progression to AD, the best accuracy was observed with the full PRS model, which includes the *APOE* region; however, this also requires the PRS component.

Note that for all the models used, the best prediction accuracy was achieved with a *p* value threshold of 0.5 for AD‐associated SNPs. The same threshold was previously reported in studies that were done on different genotyping arrays.^13^, ^14^ For the best prediction accuracy in clinical practice, PRS should be generated on a set of SNPs in a way that captures genetic liability of the whole genome.

The potential implication of these findings is that the *APOE* gene affects amyloid deposition but that much of the rest of the risk of disease is involved in the rate at which amyloid deposition causes a neurodegenerative response. Clinical trials have previously shown that there is little correlation between AD progression and accumulation of amyloid plaques, supporting a hypothesis that AD development may have 2 separated stages: amyloid dependent and amyloid independent.^33^ It is also known that the *APOE* gene influences the deposition of amyloid in the brain^34^ and that this is necessary but not sufficient for development of clinical AD. Moreover, it has been shown that neuronal loss and tangle numbers increase as AD progresses,^35^ unlike the number of amyloid plaques, which reaches its maximum^36^ with the onset of clinical symptoms.

While analysis of early onset AD firmly implicated amyloid precursor protein metabolism and Aß production in the etiology of the disease, GWASs and exome and genome sequencing have implicated with varying degrees of confidence a number of potentially biologically relevant pathways in late onset AD pathogenesis.^21^, ^22^ Of course, pathway construction is an imperfect art both because of the knowledge base used in the generation of the pathways and because proteins may have more than one function in more than one cell type. Nevertheless, it is valuable to divide polygenic risk by pathways both in terms of modeling the disease through induced pluripotent stem cell technologies (one might like to assign high or low risk by pathway) and in terms of eventually tailoring therapies to pathway deficits. To dissect AD PRS by biologically relevant gene sets, we tested pathways enriched in AD^6^ identified by IGAP. All pathways except “activation of immune response” were highly significantly associated with AD risk and amyloid deposition risk; however, most of the signal was attributed to the *APOE* region alone. Protein–lipid complex showed the strongest association with AD and amyloid deposition risk in all the analyses.

In conclusion, our results imply that *APOE* contributes to disease risk in a manner that is mechanistically different from the other genetic contributors to disease risk. We speculate that *APOE* affects amyloid deposition and that the PRS affects conversion from amyloid positivity to AD. Therefore, in the context of the amyloid cascade hypothesis, *APOE* acts prior to amyloid deposition and the remaining genetic risk factors identified through GWASs act between amyloid deposition and clinical onset of AD.

## Author Contributions

G.L., M.S., E.B., and R.S. contributed to the acquisition and data analysis; G.L., M.S., E.B., R.S., J.H., and V.E.‐P. contributed to the drafting of the manuscript or part of it; J.H., J.W., and V.E.‐P. contributed to the critical review of the manuscript; J.H., J.W., and V.E.‐P. contributed to the conception and design of the study.

## Potential Conflicts of Interest

Nothing to report.
